# Characteristics Study of In-Situ Capacitive Sensor for Monitoring Lubrication Oil Debris

**DOI:** 10.3390/s17122851

**Published:** 2017-12-08

**Authors:** Zhibin Han, Yishou Wang, Xinlin Qing

**Affiliations:** School of Aerospace Engineering, Xiamen University, Xiamen 361005, China; hanzb@stu.xmu.edu.cn (Z.H.); xinlinqing@xmu.edu.cn (X.Q.)

**Keywords:** capacitive sensor, lubrication oil debris, characteristics, engine health monitoring

## Abstract

As an essential part of engine health monitoring (EHM), online lubrication oil debris monitoring has recently received great attention for the assessment of rotating and reciprocating parts in aero-engines, due to its high integration, low cost and safe characteristics. However, it is be a challenge to find a suitable sensor operating in such a complex environment. We present an unconventional novel approach, in which a cylinder capacitive sensor is designed and integrated with the pipeline of an engine lubrication system, so that the capacitive sensor can effectively detect changes in the lubrication oil condition. In this paper, an attempt to illustrate the performance characteristics of the developed cylinder capacitive sensor is made, through an experiment system that simulates a real scenario of a lubrication oil system. The main aim of the research was to qualitatively describe the relationship between the sensor parameter and the lubrication oil debris. In addition, the effect of the temperature and flow rate of the lubrication oil on capacitance change was performed by several experiments and we figured out a compensation method. The experimental results demonstrated that the cylinder capacitive sensor can potentially be used for lubrication oil debris monitoring of the health condition of an aero-engine.

## 1. Introduction

Aircraft engine health monitoring (EHM) plays an important role in many of the nation’s key industries, including aerospace, manufacturing, and energy. Central to EHM, lubrication monitoring is essential to provide an early warning in failure progression and also extends the quantity of lubrication oil, in order to ensure engine reliability and security and reduces maintenance costs and environmental pollution.

Flowing lubrication oil can not only cool the working parts and protect metal surfaces against corrosion, but it also transports the debris/particles produced by mechanical wear, which can reflect the healthy status of the engine and its components. The wear particles in lubrication oil maintain a constant concentration and small size in normal working conditions; however, when abnormal wear occurs, the concentration and size of the particles increase and may result in overheating and component failure [1]. Therefore, the detection of lubrication oil debris is an essential and effective means for identifying an engine’s wear condition and providing a prognosis in pending failure. Generally, the types of debris can be divided into ferrous and nonferrous. It has been shown that the size and forms of debris produced in different wear states are different, as illustrated in Table 1.

Many studies from academia and industry have been made in the past decade, to detect the conditions of lubrication oil. Laboratory (offline) and in situ (online) methods are both used in metal particle analysis. The offline approaches require a physical sample of the lubrication oil collected from the lubrication oil circulation system. Therefore, a diagnostic database and a long period are needed. Currently, these offline measurement methods include ferrography [2] and spectrometric analysis [3]. In comparison, online methods normally utilize sensors installed on the engine to continuously monitor circulating lubrication oil during operation. Online methods can perform real time analysis with no request for complicated setup and long-time analysis in a laboratory. In situ monitoring of lubricant oil quality has become an important issue in today’s military, transportation and manufacturing industries [4].

Over the years, scientists and experts have developed a wide range of online lubrication oil condition methodologies that can provide continuous monitoring of an engine’s health condition. These methods include optical detection [5], photoelectric and magnetic hybrid detection [6], inductive detection [7,8], capacitance detection [9,10,11], ultrasonic/acoustic detection [12], electrical impedance detection [13], online x-ray spectrography [14], high temperature dynamic viscosity sensor [15] and electrostatic charge detection [16]. The advantages and disadvantages of all these online condition monitoring technologies have been listed in the literature [17]. Wu et al. [18] (2013) comprehensively reviewed the progress of on-line lubrication oil monitoring techniques, mainly focusing on sensor technologies, their scopes and industrial applications. The final goal of all of the above-mentioned technologies is to achieve lubrication of the oil health condition in a complex engine and solve some challenges, such as environmental compensation, compatibility with structures and electromagnetic property, high reliability and robustness, high resolution and accuracy for weak and random signals.

To remedy the limitation of a single method, many researchers have proposed some integrated approaches to play their own advantages. For example, Appleby et al. [10] (2013) combined ultrasonic, capacitance and inductance-based methods to detect lubrication oil debris contents and analyze other physical parameters associated with lubricating oil degradation. Matsumoto et al. [2] (2016) presented a hybrid approach combining scanning electron microscopy (SEM) and ferrography to observe the wear debris particles in lubricant oil during a stable wear state and an abnormal wear state. Xu et al. [12] (2015) adopted matching pursuit and quantum-behaved particle swarm optimization to extract the ultrasonic echo wave shape features that can distinguish debris with different shapes and air bubbles. Some useful apparatuses have been developed with microfluidic techniques to detect the individual particles based on different principles [19]. These devices include the resistive pulse sensor, capacitance counter sensor and inductive counter sensors [20].

Among all these means, capacitive sensors have been widely used because of the advantages of good temperature stability, simple structure, strong adaptability, good dynamic response, and noncontact measurement [21]. Although the capacitance methods have some problems, such as sensitive to lubrication oil quality, such as total acid number, water content and viscosity, they are still the most practical and effective methods in many applications [22,23]. However, further studies on the characteristics of capacitive sensors are required to improve their technology maturity. Therefore, in this article, we not only study the design of an in-situ capacitive sensor for monitoring debris of engine lubrication oil, but also explore the characteristics of capacitive sensors. The basic principle of designing is to utilize the influence of dielectric permittivity. Namely, when the debris gets into the lubrication oil and causes a change in the dielectric constant of lubrication oil, the capacitance will vary as well. Hence, we can use the capacitance change to characterize whether the lubrication oil contains debris or not. The novelty of the presented capacitance sensor can be summarized by two aspects. One is that the sensor structure is simple and characterized by two coaxial cylinders as electrodes, while conventional capacitive sensors are composed of two parallel plates as electrodes. This structure feature is beneficial as it allows easy integration with the lubrication oil pipeline. The other is that the sensing mechanism and physical model of the presented sensor is verified and validated by a simple proof-of-principle experimental system.

The aim of this work was to conduct a feasibility study on the application of the capacitive sensing principle for detecting debris in lubricant oil. The paper is organized as follows: Firstly, the theoretical model of the capacitive sensor is developed to analyze sensor characteristics. Next, the preliminary experimental results are presented and key conclusions are drawn. Finally, a compensation for temperature and flow rate is proposed.

## 2. Sensing Principle and Sensor Model

### 2.1. Cylinder Capacitive and Their Sensing Principle

**Figure 1:**
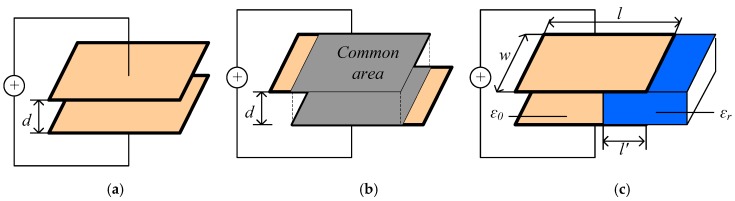
Schematic diagram of conventional capacitive sensor structure: (**a**) Variation in the distance between the plates; (**b**) Variation of the common surface; and (**c**) Change in the dielectric element.

A capacitive sensor is a device whose physical characteristics determine the value of its capacitance. These characteristics include the distance between two electrodes, the common surface, and the dielectric element, as shown in Figure 1. Generally, the materials of electrodes can be copper or iron. To the authors’ knowledge, most structures of capacitive sensors are plate-like, with the same or similar area [21]; however, these structure forms can be evolved into circular ones, for example coxial capacitive sensors [24,25] and micro-fluid capacitive sensors [26].

**Figure 2:**
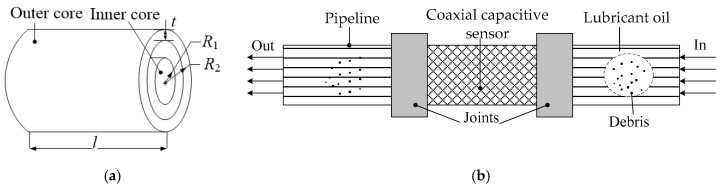
Schematic diagram of the presented capacitive sensor model: (**a**) Coaxial capacitive sensor model; (**b**) Integration scheme of the coaxial capacitive sensor and lubricant oil pipeline.

Considering the lubrication oil pipeline is small in diameter, this study presents a coaxial capacitive sensor, as shown in Figure 2a. It includes three components: the outer core, the inner core and the connector or joint. The connector is used to connect two cores and integrate with the original pipeline. The whole assembly scheme is illustrated, as shown in Figure 2b. It can be obviously seen that the presented structure is simple, and can be easily installed on the lubrication oil pipeline. The joint can be either a connecting flange or a nut with two pipelines. Lubrication oil flows through an annular space between the outer core and the inner core. If there is no debris in the lubrication oil, the dielectric constant between two poles is stable in value, which means that the measured capacitance is unchanged. When the lubrication oil contains debris, the value of the dielectric constant between two poles will change, causing change in the capacitance. The possibility of this type of capacitive sensor has been substantiated in [24]. It should be noted that the solid center conductor/inner core will cause a change in flow structure and produce turbulence when oil flows into the sensor. Since the value of Reynolds number is small, this study will not consider the effect of turbulence caused by the solid center conductor. In future investigation, we will optimize the parameters of coxial capacitance, such as the length and radius of the center conductor to minimize the influence of turbulence.

### 2.2. A Mathematical Model

The capacitance of the sensor depends on the dielectric permittivity of the medium between the pair of electrodes. For a conventional parallel capacitive sensor, it is easy to calculate the capacitance (C) between the two parallel plates (electrodes) of a capacitor, according the formula described in [21]. However, there is no formulation for the presented sensor structure, especially when debris is flowing into the capacitive sensor.

**Figure 3:**
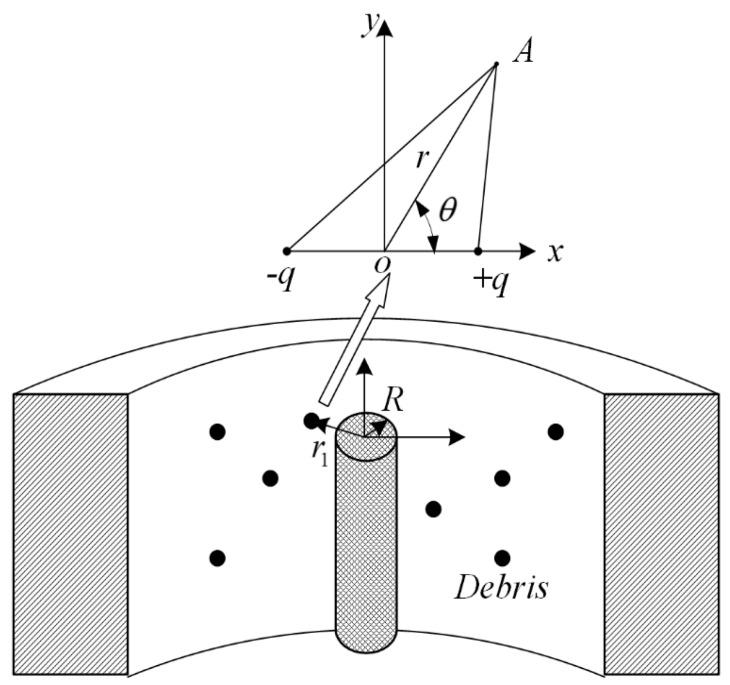
Setting up a coordinate system in a debris.

In order to establish a mathematical model of a capacitive sensor with debris flowing through, this study presents some assumptions, as follows: The debris is regarded as electric dipoles and the quantity of electric charge (Q) is much less than the capacitive sensor. Based on these assumptions, a coordinate system can be set up in debris. Figure 3 illustrates the process.

Suppose the debris particle is at point X, −q and +q represent two opposite poles. Any point A gets very close to X. Electric dipoles generate additional voltage, $V_{0}$, in A.

(1) $$V_{0}=\frac{1}{4\pi \varepsilon_{0}}\frac{p\cos\theta }{r^{2}}$$

where θ and *r* are the angle and the distance between A and an electric dipole, *p* is the particle’s electric dipole moment and *p* is in direct proportion to the quantity of electric charge, *q*, induced in the debris, so

(2) $$p=q\times r_{0}$$

where $r_{0}$ is a constant, representing the distance between two electric dipoles.

The capacitive sensor creates an original electric field and generates original voltage. The original voltage at point A is defined as *V*.

(3) $$V=\frac{Q}{2\pi \varepsilon l}\ln\frac{r_{1}}{R}$$

*Q* is the quantity of electric charge in the capacitive sensor, *R* is the radius of the sensor’s inner core, $r_{1}$ is the distance between the debris position and origin coordinates building in the sensor.

Based on Equations (1) and (3), we get Equations (4) and (5).

(4) $$C=\frac{Q}{V}=\frac{2\pi }{\ln(\frac{R_{1}}{R})}\varepsilon$$

(5) $$C_{0}=\frac{Q}{V_{0}}$$

where C is the capacitance of the sensor, *R*_1_ is the radius of the sensor outer core and $C_{0}$ means additional capacitance. Since *Q* is much larger than q, Equation (5) utilizes *Q* instead of *Q* + *q*.

Thus, the total capacitance at point A is

(6) $$C_{T}=C+C_{0}=C+\frac{Q}{V_{0}}=C+\frac{Q4\pi r^{2}\varepsilon_{0}}{p\cos\theta }=C+K\varepsilon_{0}$$

From Equations (3) and (4), we know that the capacitance of sensor *C* is a constant. Hence, it is easy to see that the total capacitance, $C_{T}$, will vary linearly with dielectric permittivity, $\varepsilon_{0}$, The expression of *K* is

(7) $$K=\frac{Q4\pi r^{2}}{p\cos\theta }$$

Equation (7) illustrates that *K* will vary with θ and *r*. Ideally, if the debris is fixed at one point, θ and *r* will become constant, that is, *K* is a constant. However, the location of the debris varies with time. In actual conditions, *K* will change in various cases, but remain a constant in one case. Experiments will verify the characteristics of *K*.

## 3. Experiment

### 3.1. Experimental Setup

**Figure 4:**
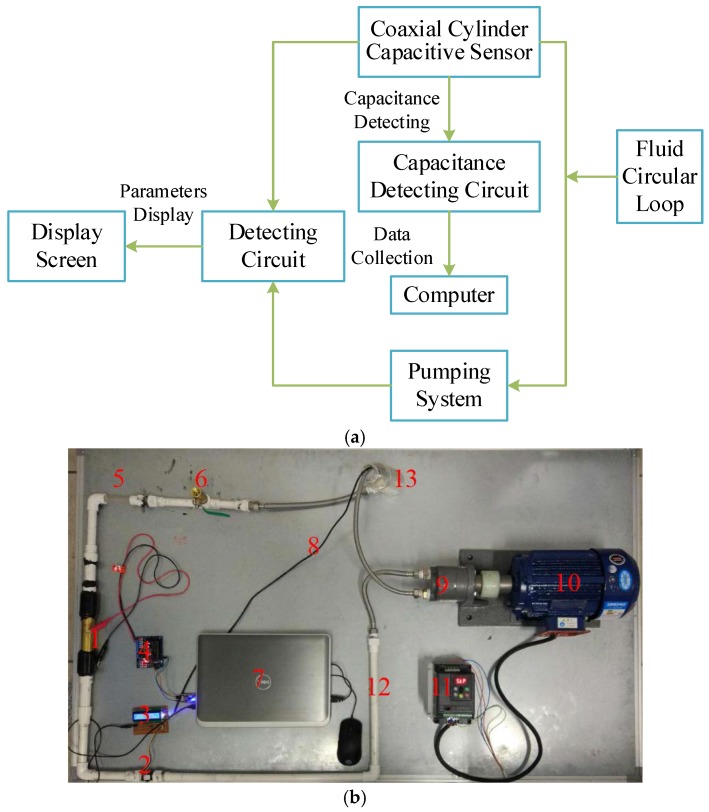
Experimental set-up: (**a**) experimental flow chart; (**b**) experimental platform.

To demonstrate the effectiveness of the capacitive sensor, a series of experiments were conducted. The experimental set-up used for the analysis of lubrication oil debris consisted of a data acquisition system, fluid circular loop, detectable object, pumping system, and temperature detecting circuit. The entire experiment set-up is illustrated in Figure 4. The values of the four parameters, as shown in Figure 2a, were set as follows: *R*_1_ = 14 mm, *R*_2_ = 4 mm, *t* = 2 mm, and *l* = 150 mm.

Figure 4a elucidates the experimental sequence and Figure 4b shows the experimental platform. Lubrication oil containing debris was loaded into one reservoir and pumped into the fluid circular loop to pass the sensor by the pump system, which was able to control the flow rate from 0 to 10,000 mL per minute, and then returned into the reservoir. The capacitive sensor, mentioned earlier, was also integrated into the loop, which is numbered 1 in Figure 4b.

The temperature of the lubrication oil affects its permittivity and thus the capacitance signal will change as well. During the operating process, the temperature of test lubrication oilcan range from 30 °C to 40 °C. To simulate real conditions of lubrication oil, we controlled the lubrication oil temperature at 30 °C with 2 °C precision. A temperature sensor probe was assembled, to measure the real time temperature of lubrication oil. All experimental components are listed in Table 1. The numbers in Table 2 correspond to those marked in Figure 4b.

### 3.2. Measurement System

The traditional data acquisition system was mainly composed of an LCR meter with a signal collection circuit, to directly acquire statistics. The LCR meter is an instrument that can measure capacitance directly. However, the data obtained from LCR meters are often fluctuating. For this reason, we adopted a new data acquisition system, which consisted of a capacitance detecting circuit and a computer. The data acquisition system is shown in Figure 4, which is numbered as 4.

When the capacitive sensor is out of service, the output signal of the circuit is a resonance frequency, *F*_1_, generated by capacitor *C*_1_ and inductor *L*_1_. Equation (8) demonstrates this process.

(8) $$F_{1}=\frac{1}{2\pi \sqrt{L_{1}C_{1}}}$$

When the capacitive sensor starts working, the output signal is changed into a resonance frequency, *F*_2_, generated by shunting capacitors (*C_x_//C*_1_) and inductor *L*_1_. Thus, we get Equation (9).

(9) $$F_{2}=\frac{1}{2\pi \sqrt{L_{1}(C_{1}+C_{x})}}$$

According Equations (8) and (9), the unknown parameter *C_x_* can be computed by Equation (10).

(10) $$C_{x}=C_{1}(\frac{F_{1}{}^{2}}{F_{2}{}^{2}}-1)$$

**Figure 5:**
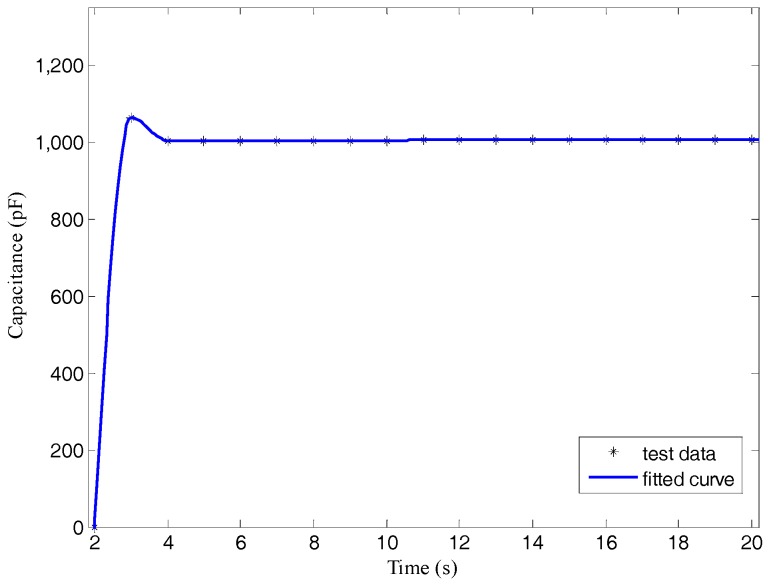
Digital signals output of a 1000 pF capacitor.

The entire process of date acquisition was composed of three main steps: collecting analog signals, converting analog signals into digital signals, and gaining digital signals. Finally, we acquired the digital signals, that is capacitance, in the computer. Taking a 1000 pF capacitor as an example, the final digital signal outputs are displayed in Figure 5.

## 4. Results and Discussion

### 4.1. Relationship between the Capacitance and the Debris Quantity

To explore the relationship between output signals of the capacitive sensor and debris quantity, a series of experiments were carried out and this section presents the experimental results. To demonstrate that the relationship between the output signal of the capacitive sensor and debris quantity is independent of the debris-carrying medium, water carrying the debris under the same conditions as lubricant oil was introduced to make the comparison. Both media-carrying debris particles were pumped from the inlet reservoir to the outlet reservoir using the pump system. In these experiments, the debris quantity ranged from 0.00 g to 0.40 g and test temperature was set up as room temperature (26.5 °C); the flow rate was set to 4210 mL per minute. All these statistics are shown in Table 3.

**Figure 6:**
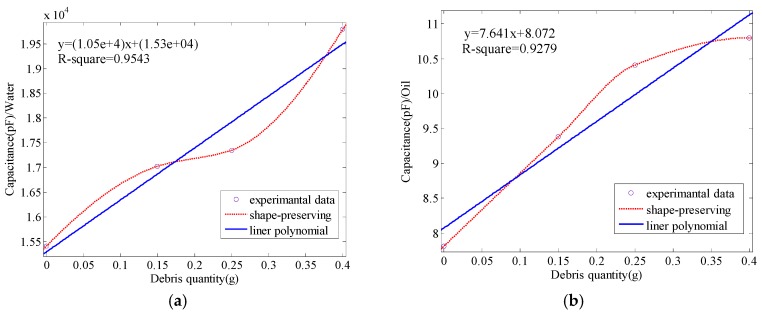
The value of capacitance change with debris quantity in water or oil: (**a**) water; (**b**) oil.

As shown in Table 3, the measured capacitance increases with a rise in debris quantity. To illustrate the relationship more clearly, the data in Table 3 are graphically shown in Figure 6.

Based on the least-squares method, Figure 6 presents the linear relationship between capacitance and debris quantity. Moreover, both the R-square goodness of fits are above 0.92, which means the linearity is highly trustworthy. What is more, the fitting equations have been shown in Figure 6. Different quantities of debris can be seen as the dielectric permittivity varies. When the oil or water with debris flows into the capacitive sensor, the medium in the sensor will change based on the purity of water or oil, that is, the dielectric permittivity of pure water or oil is different from the dielectric permittivity of water or oil with debris. Furthermore, changes in the amount of debris will cause changes in the dielectric permittivity of the medium in the sensor. Equation (6) claims that the capacitance will vary linearly with dielectric permittivity. It is clear that the experimental results, as shown in Figure 6, verify the deduction in Section 2. The size of debris flowing into the capacitive sensor is as small as several micrometers when the cosθ in Equation (7) is almost equal to 1. When the debris is moving with the water or water, the position of the debris can be treated as fixed at one point because of the large flow rate and short distance between the outer core and inner core. The parameter can be seen as a constant and the *K* illustrated in Equation (7) becomes constant. However, different mediums have a different *Q* in Equation (7) and a different *C* in Equation (6)—this is why the fitting equations are not the same in Figure 6a,b.

### 4.2. Relationship between the Capacitance and the Oil Temperature

Section 4.1 showed the functionality of the capacitive sensor, but the experiments undertaken for debris quantity have their limitations. While the temperature and the flow rate were set to be constant in all experiments conducted in this study, the temperature and the flow rate will certainly not maintain fixed values in practice. Hence, this section investigates the temperature influence, while the flow rate is constant and set as 4210 mL/min, and there is no debris in the oil. Experiments were performed and all data is listed in Table 4.

**Figure 7:**
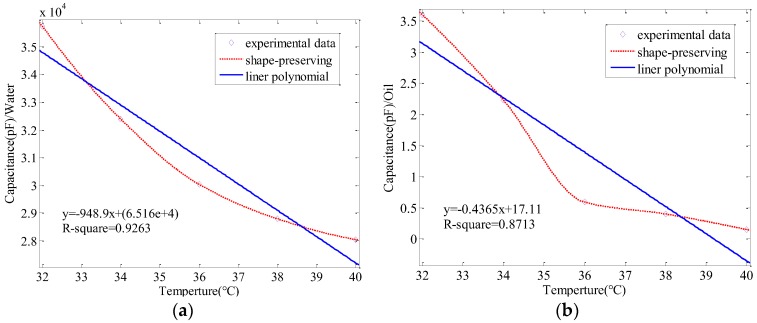
The value of capacitance change with temperature in water or oil: (**a**) water; (**b**) oil.

Table 4 shows that capacitance increases with a rise in temperature. To obviously illustrate the relationship between the capacitance and the temperature, the data in Table 4 are plotted in Figure 7. It is easy to see the relationship from the fitting curve.

As shown in Figure 7, the relationship between capacitance and temperature demonstrates the same pattern in both media. With an increase in temperature, the measured capacitance will decrease, since the dielectric permittivity of water or oil will increase with the temperature rise. The nature of the relationship between the temperature and the measured capacitance is the relationship between the dielectric permittivity and the measured capacitance, which verifies Equation (6). In fact, these experimental results are also consistent with the Equation (4), that is, the expression of capacitance without any debris. The R-square goodness of fit in Figure 7a is equal to 0.9263, so the linearity is highly trusted. In Figure 7b, the R-square goodness of fit is 0.8713. This value is not as good as the experiments with water, but still can support the linearity. However, the experimental group has its limitations. In the process of date acquisition, we needed to convert analog signals into digital signals so some noise will mix into and cause greater experimental value. In spite of the existence of this limitation, the tendency shown in these experiments is actually valid, which is what we are most interested in this article.

### 4.3. Relationship between the Capacitance and the Flow Rate

The flow rate is another crucial factor that affects the measured capacitance. Similar to temperature, the flow rate causes inaccuracy in experimental results. To study the influence of the flow rate, a similar method to that described in Section 4.2 was adopted. The temperature was constant and set as 25.8 °C; there was no debris in the medium. Data collected from experiments are shown in Table 5.

**Figure 8:**
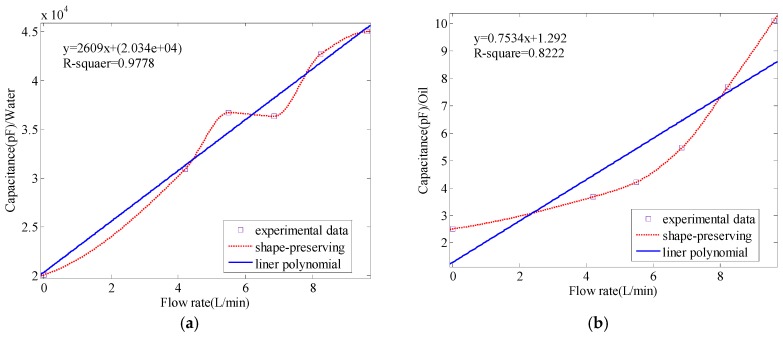
The value of capacitance change with flow rates in water or oil: (**a**) water; (**b**) oil.

In order to display the trends of all these data, we plotted the curve in Figure 8. Apparently, Figure 8 reveals the linear relationship as well.

To demonstrate the linear relationship in Figure 8, we discuss Equation (6) again. Generally, the flow rate will not affect the dielectric permittivity, but it will cause the production of static electricity during the process of flowing. The production of static electricity changes the quantity of electric charge (*Q*) in Equation (6). Thus, the dielectric permittivity in Equation (6) remains constant and the parameter *Q* is treated as an independent variable; the linear relationship still holds. Hence, based on Equation (6), the measured capacitance varies linearly with the flow rate. According to Figure 8a, the R-square goodness of fit is above 0.97. This means the linearity is highly trustworthy. In Figure 8b, the R-square goodness of fit is about 0.82. This linearity is not so high, but acceptable. If we consider the tendency in Figure 8b, we find that the curve, which decreases firstly and increases latterly, is like a wave. Given that there are more points in Figure 8b, the curve will begin to decrease again and show a similar trend as the curve in Figure 8a. Thus, with more data points in Figure 8b, the linearity will improve.

## 5. Compensation Method

Section 4.2 and Section 4.3 have elucidated the influences of temperature and flow rate. In the actual oil debris detection, the temperature and the flow rate are often varied; this will be detrimental to experimental results and affect judgments about the content of oil debris. Hence, this section proposes a compensation method.

If we assume the relationship between the capacitance and the temperature, the flow rate and the debris quantity as the following:

(11) $$C_{v}=f(v)$$

(12) $$FC_{t}=g(t)$$

(13) $$C_{m}=h(m)$$

If the flow rate, the temperature and the debris quantity are changed into *v* + *Δv*, *t* + *Δt* and *m* + *Δm*; the corresponding change in the capacitance can be expressed as follows:

(14) $$\Delta C_{v}=f(v+\Delta v)-f(v)$$

(15) $$\Delta C_{t}=g(t+\Delta t)-g(t)$$

(16) $$\Delta C_{m}=h(m+\Delta m)-h(m)$$

Most of the time, the quantity of wear debris from the engine is unknown. What we can measure is the flow rate change, *Δv*, the temperature change, *Δt*, and the capacitance change, *ΔC*. As stated in Section 4, the relationship between the measured capacitance and the temperature, the flow rate and the debris quantity can be simplified as a linear relationship in the case of a single parameter. Therefore, the capacitance change caused by the debris can be calculated according to Equation (14) to Equation (16).

(17) $$\Delta C_{m}=\Delta C-\Delta C_{v}-\Delta C_{t}$$

Combining Equations (13) and (17), we can acquire the debris quantity using the capacitance change, so the goal of detecting oil debris has come to realization. A set of experimental data was utilized to justify the accuracy of this compensation method. 

Because the temperature, 36 °C, is the middle value in temperature experiments, we chose 36 °C as a reference temperature to verify the compensation method. Thus, Table 3 presents the conditions for a flow rate of 4210 mL/min and no debris, to serve as a baseline. Next, the capacitance was measured when there was 0.4 g debris in the oil with a 9610 mL/min flow rate and a temperature of 40 °C. This data is regarded as an experimental group, as shown in Table 5.

Hence, according to Table 4, the capacitance change, *ΔC_v_*, caused by the flow rate is calculated as:

(18) $$\Delta C_{v}=10.11-3.68=6.43(\mathrm{pF})$$

Similarly, according to Table 3, the capacitance change, *ΔC_t_*, caused by the temperature is calculated as:

(19) $$\Delta C_{t}=3.60-0.59=3.01(\mathrm{pF})$$

The difference in capacitance resulting from changing the experimental condition, *ΔC*, is:

(20) ΔC=13.36−0.59=12.77(pF)

These data are listed in Table 6. The capacitance change, *ΔC_m_*, caused by the debris, can be calculated by Equation (17).

According to Table 3, the change of capacitance caused by debris, *ΔC_m_*, can be obtained from:

(21) $$\Delta C_{m}=10.80-7.81=2.99(\mathrm{pF})$$

The error between the actual value and the calculated value is:

(22) $$\frac{3.33-2.99}{2.99}\times 100\%\approx 11.37\%$$

Considering experiments were conducted in an open environment with large random noises, the error 11.37% can be accepted. This case study verifies the possibility of compensation method for eliminating the influences of the temperature and the flow rate.

## 6. Conclusions

This paper presents a capacitive sensor structure, based on two coaxial cylinders, which is suitable for in-situ monitoring of debris in lubrication oil, and explores its characteristics. Meanwhile, this paper developed a mathematical model to describe the sensing mechanism and relative sensor features. The model proves that capacitance varies linearly with dielectric permittivity. To illustrate the relationship between debris and the capacitive sensor and verify the mathematical model, an experimental device was set up. The experiment verified that the proposed sensor configuration can characterize the debris using capacitance values. It was found that the capacitance values increased almost linearly when the debris quantity increased. Furthermore, a similar pattern was found when the temperature or the flow rate changed. These experimental results coincided with the mathematical model.

In order to eliminate the influences caused by the temperature and the flow rate, this study proposed a compensation method. The method is based on the linear characteristics exhibited by the capacitive sensor and has an acceptable accurancy. However, lots of further work is in order to apply the presented method into the application. In particular, it is necessary to optimize the design parameters of the sensor to eliminate the effect of turbulence, as well as considering integration with a real engine and a more accurate compensation method.

## Figures and Tables

**Table 1:** The forms of debris produced under different wear and tear.

Debris Types	Size in Direction of Long Axis/μm	Form Factor (Length:Thickness)
Debris of normal wear	<15	10:1
	<5	not considered
Debris of serious wear	>15	>5:1 but <30:1
Peeling piece	>5	<5:1
Laminar particle	>15	>30:1

**Table 2:** Experimental platform components.

Number	Component	Type
1	Cylinder capacitive sensor	φ30 mm × φ25 mm × 150 mm
2	Flow sensor	YF-S201C
3	Temperature detecting circuit	×
4	Capacitance detecting circuit	×
5	Transparent tube	×
6	Valve	×
7	Computer	×
8	Temperature sensor probe	DS18B20
9	Pump	CB-B32
10	Three-phase motor	YE2-90L-4
11	Converter	KZ100
12	PVC tube	DN15, DN20
13	Reservoir	×

× means no special type.

**Table 3:** Capacitance of different debris quantity in water or lubrication oil (Temperature: 26.5 °C; Flow rate: 4210 mL/min).

Debris Quantity (g)	Capacitance (pF)/Water	Capacitance (pF)/Oil
0.00	15,412.13	7.81
0.15	17,028.28	9.38
0.25	17,351.50	10.41
0.40	19,791.48	10.80

**Table 4:** Capacitance of different temperatures in water or oil (no debris; flow rate: 4210 mL/min).

Temperature (°C)	Capacitance (pF)/Water	Capacitance (pF)/Oil
40.0	28,043.91	0.15
38.0	28,793.69	0.40
36.0	30,034.88	0.59
34.0	32,391.91	2.23
32.0	35,733.86	3.60

**Table 5:** Capacitance of different flow rates in water or oil (no debris; temperature: 25.8 °C).

Flow Rate (L/min)	Capacitance (pF)/Water	Capacitance (pF)/Oil
0.00	20,097.82	2.51
4.21	30,934.81	3.68
5.49	36,710.99	4.21
6.86	36,344.96	5.47
8.23	42,695.96	7.69
9.61	45,007.76	10.11

**Table 6:** The experimental temperature and flow rate compensation method.

Group	Flow (L/min)	Temperature (°C)	Debris Quantity (g)	Capacitance (pF)	*ΔC_v_* (pF)	*ΔC_t_* (pF)	*ΔC* (pF)	*ΔC_m_* (pF)
Baseline	4.21	36.0	0.00	0.59	6.43	3.01	12.77	3.33
Experiment	9.61	40.0	0.40	13.36				

## Nomenclature

*EHM*	Engine health monitoring
*Q*	Quantity of electric charge
*V*_0_	Additional voltage
*θ*	Angle between A and electric dipole
*r*	Distance between A and electric dipole
*p*	Particle’s electric dipole moment
*q*	Quantity of electric charge induced in the debris
*r*_0_	Distance between two electric dipole
*r*_1_	Distance between debris position and origin coordinates
*V*	Original voltage at point A
*R*	Radius of sensor inner core
*R*_1_	Radius of sensor outer core
*C*	Capacitance of sensor
*C*_0_	Additional capacitance
*l*	Length of sensor
*ε*	Dielectric permittivity
*F*	Resonance frequency

## References

[1] Mauntz M.R., Gegner J., Kuipers U., Klingau S. (2013). A sensor system for online oil condition monitoring of operating components. Tribol. Fundam. Adv..

[2] Matsumoto K., Tokunaga T., Kawabata M. (2016). Engine Seizure Monitoring System Using Wear Debris Analysis and Particle Measurement.

[3] Guan L., Feng X.L., Xiong G., Xie J.A. (2011). Application of Dielectric Spectroscopy for Engine Lubricating Oil Degradation Monitoring. Sens. Actuators A.

[4] Jaw L.C. (2005). Recent advancements in aircraft engine health management (EHM) technologies and recommendations for the next step. ASME Proc..

[5] Iwai Y., Honda T., Miyajima T., Yoshinaga S., Higashi M., Fuwa Y. (2010). Quantitative estimation of wear amounts by real time measurement of wear debris in lubricating oil. Tribol. Int..

[6] Kuo W.F., Chiou Y.C., Lee R.T. (1997). Fundamental characteristics of wear particle deposition measurement by an improved on-line ferrographic analyzer. Wear.

[7] Wu Y., Zhang H., Zeng L., Chen H., Sun Y. (2016). Determination of metal particles in oil using a microfluidic chip-based inductive sensor. Instrum. Sci. Technol..

[8] Hong W., Wang S., Tomovic M.M., Liu H., Wang X. (2015). A new debris sensor based on dual excitation sources for online debris monitoring. Meas. Sci. Technol..

[9] Minasamudram R., Agarwal P., Venkateswaran P. Simulation of a Capacitive Sensor for Wear Metal Analysis of Industrial Oils. Proceedings of the COMSOL Conference.

[10] Appleby M., Choy F., Du I., Zhe Z. (2013). Oil debris and viscosity monitoring using ultrasonic and capacitance/inductance measurements. Lubr. Sci..

[11] Ding X.Y., Zheng H., Jian W.X. Research on Capacitive Sensor for Online Oil Monitoring. Proceedings of the Prognostics and System Health Management Conference (PHM-Shenzhen).

[12] Xu C., Zhang P., Wang H., Li Y., Lv C. (2015). Ultrasonic echo waveshape features extraction based on QPSO-matching pursuit for online wear debris discrimination. Mech. Syst. Signal Process..

[13] Itomi S. (2006). Oil Condition Sensor. U.S. Patent.

[14] Kayani S.A. Using combined XRD-XRF analysis to identify meteorite ablation debris. Proceedings of the International Conference on Emerging Technologies.

[15] Brouwer M.D., Gupta L.A., Sadeghi F., Peroulis D., Adams D. (2012). High temperature dynamic viscosity sensor for engine oil applications. Sens. Actuators A.

[16] Powrie H. Use of electrostatic technology for aero engine oil system monitoring. Proceedings of the IEEE Aerospace Conference Proceedings.

[17] Du L., Zhe J. (2011). On-line wear debris detection in lubricating oil for condition based health monitoring of rotary machinery. Recent Patents on Electrical & Electronic Engineering (Formerly Recent Patents on Electrical Engineering).

[18] Wu T., Wu H., Du Y., Peng Z. (2013). Progress and trend of sensor technology for on-line oil monitoring. Sci. China Technol. Sci..

[19] Zhang H.P., Chon C.H., Pan X.X., Li Q.Q. (2009). Methods for Counting Particles in Microfluidic Applications. Microfluid. Nanofluid..

[20] Du L., Zhe J., Carletta J.E., Veillette R.J. (2010). Inductive Coulter Counting: Detection and Differentiation of Metal Wear Particles in Lubricant. Smart Mater. Struct..

[21] Stevan S.L., Paiter L., Galvão J.R., Chaves E.S. (2015). Sensor and Methodology for Dielectric Analysis of Vegetal Oils Submitted to Thermal Stress. Sensors.

[22] Aslam M.Z., Tang T.B. (2014). A High Resolution Capacitive Sensing System for the Measurement of Water Content in Crude Oil. Sensors.

[23] Dong T., Barbosa C. (2015). Capacitance Variation Induced by Microfluidic Two-Phase Flow across Insulated Interdigital Electrodes in Lab-On-Chip Devices. Sensors.

[24] Wang Y., Han Z., Liang D., Qing X. Study on In-situ Capacitive Sensor for Monitoring Engine Lubricant Oil Debris. Proceedings of the 8th European Workshop On Structural Health Monitoring (EWSHM 2016).

[25] Geraets J.J.M., Borst J.C. (1988). A capacitance sensor for two-phase void fraction measurement and flow pattern identification. Int. J. Multiph. Flow.

[26] Du L. (2012). A Multichannel Oil Debris Sensor for Online Health Monitoring of Rotating Machinery.

